# Supplementary material for: Identification and functional analysis of non-coding regulatory small RNA FenSr3 in Bacillus amyloliquefaciens LPB-18
Source: PeerJ. 2023 May 15;11:e15236. doi: 10.7717/peerj.15236 (PMC10194069; doi:10.7717/peerj.15236)
Supplement: Supplemental Information 4 [file peerj-11-15236-s004.zip › KO/CK-vs-T1_map/map05169.html]

KEGG PATHWAY: Epstein-Barr virus infection - Reference pathway


|  |  |
| --- | --- |
| **Epstein-Barr virus infection - Reference pathway** |  |

[
Pathway menu
| Organism menu
| Pathway entry
| Show description
| User data mapping
]

Epstein-Barr virus (EBV) is a ubiquitous human herpesvirus that is associated with oncogenesis. EBV infection to primary human B lymphocytes leads to induction of EBV-specific HLA-restricted cytotoxic T cells, causing infectious mononucleosis during adolescence. Primary infection with EBV is followed by latent infection in which the reservoir B cells are immortalized. EBV tumorigenic potential in Burkitt lymphoma, nasopharyngeal carcinoma, Hodgkin lymphoma, and post-transplant B cell lymphomas has been well-documented. Distinct forms of EBV latency and patterns of latent gene expression can contribute to the different tumours.

|  |  |  |
| --- | --- | --- |
| Reference pathway | 184% 150% 122% 100% 82% 67% 55% | 图片下载 |
